# Ocular Cell Lines and Genotoxicity Assessment

**DOI:** 10.3390/ijerph17062046

**Published:** 2020-03-19

**Authors:** Eliana B. Souto, Joana R. Campos, Raquel Da Ana, Carlos Martins-Gomes, Amélia M. Silva, Selma B. Souto, Massimo Lucarini, Alessandra Durazzo, Antonello Santini

**Affiliations:** 1 Department of Pharmaceutical Technology, Faculty of Pharmacy, University of Coimbra, Pólo das Ciências da Saúde, Azinhaga de Santa Comba, 3000-548 Coimbra, Portugal; joanacampos92@gmail.com (J.R.C.); quele.ana@gmail.com (R.D.A.); 2 CEB-Centre of Biological Engineering, University of Minho, Campus de Gualtar 4710-057 Braga, Portugal; 3 Department of Biology and Environment, University of Trás-os-Montes e Alto Douro, UTAD, Quinta de Prados, P-5001-801 Vila Real, Portugal; camgomes@utad.pt (C.M.-G.); amsilva@utad.pt (A.M.S.); 4 Centre for Research and Technology of Agro-Environmental and Biological Sciences, CITAB, UTAD, Quinta de Prados, P-5001-801 Vila Real, Portugal; 5 Department of Endocrinology of Hospital de São João, Alameda Prof. Hernâni Monteiro, 4200–319 Porto, Portugal; sbsouto.md@gmail.com; 6 CREA-Research Centre for Food and Nutrition, Via Ardeatina 546, 00178 Rome, Italy; massimo.lucarini@crea.gov.it (M.L.); alessandra.durazzo@crea.gov.it (A.D.); 7 Department of Pharmacy, University of Napoli Federico II, Via D. Montesano 49, 80131 Napoli, Italy

**Keywords:** ocular cells lines, comet assay, DNA damage, epithelial cells

## Abstract

Genotoxicity screening tests aim to evaluate if and to what extent a compound in contact with the human body (e.g., a drug molecule, a compound from the environment) interacts with DNA. The comet assay is a sensitive method used to predict the risk of DNA damage in individual cells, as it quantifies the tape breaks, being the alkaline version (pH > 13) the most commonly used in the laboratory. Epithelial cells serve as biomatrices in genotoxicity assessments. As ca. 80% of solid cancers are of epithelial origin, the quantification of the DNA damage upon exposure of epithelial cells to a drug or drug formulation becomes relevant. Comet assays run in epithelial cells also have clinical applications in human biomonitoring, which assesses whether and to what extent is the human body exposed to environmental genotoxic compounds and how such exposure changes over time. Ocular mucosa is particularly exposed to environmental assaults. This review summarizes the published data on the genotoxicity assessment in estimating DNA damage in epithelial cells with a special focus on ocular cell lines. General comet assay procedures for ex vivo and in vivo epithelium samples are also described.

## 1. Introduction

The human eye is a sensitive organ with a unique and complex anatomy and physiology, posing interesting challenges as a drug delivery route. The tear film and conjunctiva are the first obstacles that need to be overcome for a drug to penetrate the eye. Its anatomic barriers (e.g., several layers of cornea, sclera, and retina) together with the blood–aqueous (BAB) and blood–retinal (BRB) barriers, choroidal and conjunctival blood flows also compromise the delivery of drugs into deeper tissues [1,2,3]. For the treatment of eye diseases, topical administration of a drug formulation onto the ocular mucosa is preferential over systemic administration, in which most cases require an injection that is less compliant and has a higher risk of infection. Before reaching the anatomical barrier of the cornea, any drug administered onto the ocular surface will firstly overcome the precorneal barriers. Several advantages can be highlighted in topical ocular drug delivery, namely: (i) it is needle-free, not requiring the assistance of trained personnel for administration, which improves patient compliance compared to other invasive routes; (ii) improved bioavailability of hydrophilic and low molecular weight drugs; (iii) the large absorption surface area and high vascularization offers faster drug absorption and earlier onset of action; (iv) effectiveness in emergency therapy; and (v) circumvents the hepatic first-pass metabolism, requiring less drug compared to oral administration. However, the topical eye administration also poses some disadvantages, such as: (i) the limited permeability of the cornea, resulting in low drug absorption; (ii) part of the administered dose is drained into the tear duct, causing undesired systemic side effects; and (iii) a frequent dosing regimen is usually needed because of the short duration of the therapeutic effect due to the fast clearance of the drug from the eye because of tear flow and blinking [4,5]. To overcome the identified limitations, drug delivery systems have been used to improve the bioavailability of the drugs when administered topically onto the eye mucosa. Screening for the risk of genotoxicity of these drug delivery systems in ocular cell lines is a first step in the selection of the most promising systems.

Genotoxic agents have metabolic biological activity and are able to change information encoded in deoxyribonucleic acid (DNA). This can occur when exposure to a toxic agent changes the structure or content of chromosomes (clastogenicity) or the sequence of DNA base pairs (mutagenicity). Genotoxic effects can appear in lower concentrations of substances and influence the reproduction, embryonic life, development, growth, and survival of organisms. DNA changes are also associated with carcinogenesis and hereditary defects (mutations, teratogenesis and genetic background pathologies) [6]. Genotoxicity assays are used to screen mutations in DNA and chromosomal changes induced by a drug or a formulation. As cancer may also be the result of genetic mutations, the ability of a compound to cause damage to genes is sometimes not clear [7,8].

In vitro and in vivo genotoxicity assays are fast, economically accessible, efficient in determining the ability of the new compound or new drug formulation to interact with DNA (and cause mutations, chromosome damage or affect the ability to repair DNA), and can be used as an indicator of carcinogenicity [9]. As these in vitro and in vivo assays are very simple, they can be useful in the first genotoxicity screening a new drug formulation [10]. 

The eye lens accumulates new cell layers over the existing ones which means that a chronological record of cells is kept over the course of life. The substance of the lens is composed of lens fibers; the cytoplasm of these latter is highly enriched in proteins. Over time, these proteins undergo structural changes either by natural aging or due to a systemic disease, some of which can lead to opacification. Aging is a major risk factor for the onset of several chronic diseases, and any structural changes may end up in the loss of capacities, including vision. Besides, DNA is particularly sensitive to reactive oxygen species (ROS); these can be neutralized using nutraceuticals with antioxidant properties [11,12,13,14,15,16,17,18,19,20,21,22,23,24,25], which protect DNA, promote its repair, and reduce the risk of age-related diseases. Single-cell gel (SCG) electrophoresis is a sensitive genotoxicological test that evaluates DNA damage in individual cells and enables the quantification of tape breaks [26,27,28]. In comparison to other techniques, such as 53BP1/γH2AX focus formation assays, the SCG electrophoresis assay shows the effect of genotoxic agents on the physical status of genomic DNA and can readily detect eventual antioxidant effects on DNA double-strand break formation [29]. This technique was developed by Östling and Johanson when working with micro-gel DNA electrophoresis [30], and by Singh et al. who, through the use of an alkaline solution, demonstrated its high sensitivity [31]. Over the years, this single-cell gel electrophoresis technique has been extensively modified and validated, now commonly referred to as the comet assay [32]. The advantages of the SCG test include its precision, reproducibility, simplicity, and fast performance [26,33]. It is sensitive for DNA damage detection, it allows for data analysis of individual cells, it requires only a very small amount of sample to be tested and is applicable to all eukaryote cells. Any cell type with a nucleus can be tested and, besides that, this assay requires only a small number of cells. It allows for image analysis, thus facilitating the measurements, while the costs are also extremely low. The comet assay is becoming a standard laboratory approach for the screening of formulations’ risk in inducing DNA damage [26,34]. In this review, the concept behind the comet assay and its implementation in ocular epithelial cells are discussed, summarizing published data that focus on its use in the evaluation of DNA damage.

## 2. The Anatomy and Physiology of the Human Eye

**Figure 1:**
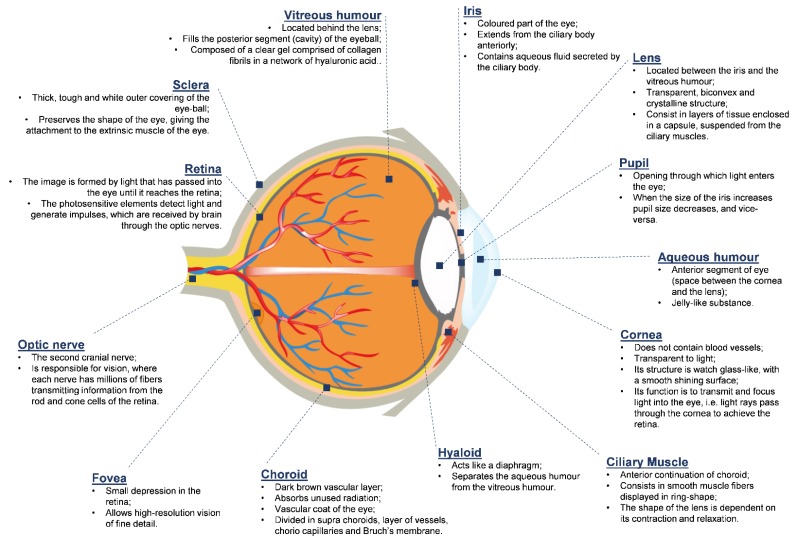
Anatomical and physiological features of the eye structure.

The human eye is a complex structure that collects information about the surroundings. The eye—an essential sensory organ—allows visualization through its ability to stimulate the nervous system by refracting light, thus producing a focused image [4]. The ocular globe with its complex anatomy is composed of different structures. The eyeball is so named because of its globe shape, which is confined inside a bony cavity and protected by the eyelids. It is divided into three layers: (i) the outermost layer, consisting of the cornea and the sclera; (ii) the anterior middle layer, consisting of the iris, choroid and ciliary body; and (iii) the inner layer composed of the retina, which is an extension of the central nervous system (CNS) [35]. Figure 1 describes the main anatomical and physiological features of the eye structure.

## 3. Comet Assay

**Figure 2:**
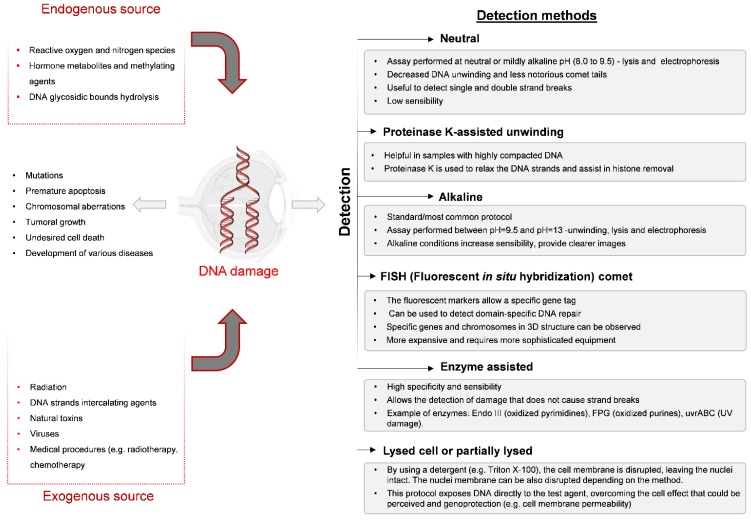
Sources of DNA damage and detection methods in comet assays.

Several ocular cell lines are available for the evaluation of the drug response and risk of DNA damage. Table 1 summarizes their main features. The integrity of DNA is instrumental to health, but the molecule is vulnerable to ROS, which induce its damage. The guidelines for the correct use of the comet assay in genetic toxicology were launched at the International Workshop on Genotoxicity Test Procedures (IWGTP) and concluded that the alkaline version (pH > 13) is the best version of this assay for identifying genotoxic activity [31,36]. This comet assay can detect DNA single-strand breaks (SSB), alkali labile sites (ALS), DNA-DNA/DNA-protein cross-linking, and SSB associated with incomplete excision repair sites [36]. Figure 2 summarizes the endogenous and exogenous sources of DNA damage and the different approaches used in the comet assay for its detection.

SCG electrophoresis or the comet assay allow the mapping of DNA damage in human cells in vivo, not only from environmental and occupational exposure but also for therapeutic purposes. Due to its versatility, this experimental test allows the exploration of the use of different cell types to assess DNA damage (e.g., epithelial cells). As most solid cancers have an epithelial origin, it becomes relevant to screen for the risk of DNA damage in these types of cells [36]. Epithelial cells are known to have common structural features but have diverse functions due to many specialized adaptations [36,57,58,59]. The human biomonitoring comet assay studies using epithelial cells improved the protocols and methodological conditions. It is not possible to generate a common protocol for all types of epithelial cells. In this section, comet assay protocols using different ocular cell lines are described. A summary of the comet assay protocols in epithelial cells are depicted in Table 2. Table 3 provides a description of methods for isolation and separation of cells from different ocular tissues to be used in comet assays.

### 3.1. Lens Epithelial Cells

Sorte et al. proposed the removal of the cells of the cornea and anterior chamber, maintaining of the samples in minimum essential media, and a single rhexis placed in Eagle’s minimal essential medium (DMEM) containing 10% fetal bovine serum (FBS) [60]. For sample preparation, the same authors proposed the preparation of a cell suspension using mechanical stirring in 50 μL of PBS for 10–15 min at 4 ℃ to drop lens epithelial cells from the lens capsule. For the comet assay, the procedure used by Singh et al. with modifications is then applied [31]. Cells in the second agarose layer are then embedded by mixing equal volumes of the cell suspension with 2% low melting point agarose (LMPA).

For sampling and storage, Zhang et al. proposed the removal of the anterior chambers and placed them in DMEM containing 15% FBS. Samples are collected within 30 min. The authors proposed the suspending of single cells by sampling lens epithelium several times. After discarding the capsules, the cell suspensions are centrifuged at 200 × g for 5 min at 4 ℃, the supernatant is discarded, and the cells are resuspended in PBS. To run the comet assay, the cell suspension is mixed with 0.75% LMPA onto pre-coated slides with 0.75% normal melting point agarose (NMPA) [36,61].

Ringen et al. proposed the removal of the anterior chamber and the immediate placement of the tissue samples in DMEM/F12 containing 15% FBS, following the sample analysis in the same medium at 37 ℃ in the presence of 5% CO_2_ [62]. For the sample preparation for the comet assay, the authors also proposed the suspending of single cells by sampling the lens epithelium several times. After the discard of the capsule, the cell suspensions are also centrifuged at 200 × g for 5 min at 4 ℃, the supernatant is discarded and the cells are resuspended in PBS. For the comet assay, the cell suspension is mixed with 1% LMPA and some drops placed onto a dried glass slide pre-coated with agarose. The authors also describe the enzymatic treatment using lesion-specific enzymes to detect specific types of DNA damage [36].

### 3.2. Corneal Cells

Haug et al. stored the corneas in Optisol GS at 4 ℃ prior to transplantation, collecting the remaining corneoscleral rims [63]. In this study, 10 rims were needed for the comet assay. For the sample preparation, the authors carted off the epithelium by scraping on ice before gentle pipetting, followed by centrifugation and resuspension of the cells in PBS. For the comet assay and enzymatic treatment, lesion-specific enzymes were utilized for the detection of specific types of DNA damage [36].

Lorenzo et al. utilized human corneoscleral tissue (in this case obtained from rings after penetrating keratoplasty). The corneolimbal rings were transferred to DMEM/F12 containing dishes, in which the peripheral sclera and cornea were trimmed off. These rings were split into 12 samples which were then washed in Hanks balanced salt solution (HBSS) in the absence of Ca^2+^ and Mg^2+^ at room temperature. For sample preparation, the samples from each ring were incubated at 37 ℃ in a humid atmosphere containing 5% CO_2_ in pre-equilibrated trypsin in HBSS containing EDTA-4Na in the absence of Ca^2+^ and Mg^2+^. An equal amount of serum-containing growth medium (DMEM/F12) was added to terminate enzyme activity by the end of the incubation period. Cells were gently dispersed and the dissociated cells transferred from each well in media/enzymatic solution to tubes on ice [36,64]. To perform the comet assay and enzymatic treatment, the procedure developed by Azqueta et al. with modifications was been applied [63,64,65].

### 3.3. Exfoliated Tear Duct Cells

In this type of comet assay, for the sampling protocol and sample storage, nasal brushing is carried out to obtain the tears utilizing a capillary tube from the inner nasal angle of the eye, stimulating the olfactory bulb. The samples are maintained in the capillary tubes at room temperature prior to performing the comet procedure. 

**Table 2:** Summary of the comet assay protocols in epithelial cells.

Cell Type	Previous Treatment	Lysis Solution	Electrophoresis Conditions	Neutralization Solution	Staining	Ref.
Corneal (porcine)	Cells, suspended in 1% Type VII low gelling point agarose, were transferred to a precoated slide with 1% standard agarose	pH = 10 Triton X-100 2.5 M NaCl 0.1 M EDTA 10 mM Tris	Alkaline pH (not specified) 300 mM NaOH 1 mM EDTA 25 V/300 mA 20 min	pH = 7.5 0.5 M Tris	Ethidium bromide	[66]
Cornea (human)	Cells, suspended in 0.65% low melting point agarose, were transferred to a slide coated with 0.65% normal melting point agarose	pH = 10 2.5 M NaCl 100 mM EDTA 10 mM Tris 1% Triton X-100 10% DMSO	pH > 13 300 mM NaOH 1 mM EDTA pH > 13 20 V/300 mA 20 min	pH = 7.5 0.4 M Tris	Ethidium bromide	[67]
Cornea and retina (rat)	Cells, suspended in 0.5% low melting point agarose, were transferred to a precoated slide with 1% agarose (type I)	pH = 10 2.5 M NaCl 100 mM EDTA 10 mM Tris 1% N-laurylsarcosin 10% DMSO 1% Triton X-100	pH > 13 300 mM NaOH 1 mM EDTA 0.78 V/cm 40–60 min	pH = 7.5 0.4 M Tris	Propidium iodide	[68]
Cornea (human)	Cells, suspended in 0.65% low melting point agarose, were transferred to a slide coated with 0.65% normal melting point agarose	pH = 10 2.5 M NaCl 100 mM EDTA 10 mM Tris 1% Triton X-100 10% DMSO	pH>13 300 mM NaOH 1 mM EDTA 20 V/300 mA 20 min	pH = 7.5 0.4 M Tris	Ethidium bromide	[69]
Lens (human)	Cells, suspended in 0.6% low melting point agarose, were transferred to a slide coated with normal melting point agarose	pH = 10 2.5 M NaCl 10 mM Tris 100 mM Na_2_ EDTA 1% Triton X-100	Alkaline pH (not specified) 300 mM NaOH 1 mM EDTA 20 V 20 min	pH = 7.5 0.4 M Tris	Ethidium bromide	[70]
Lens (human)	Cells, suspended in phosphate buffer saline and 1% low melting point agarose, were transferred to a slide precoated with agarose (not specified)	pH = 10 2.5 M NaCl 10 mM Tris 100 mM EDTA 1% Triton X-100	pH = 13 300 mM NaOH 1 mM EDTA 1.4 V/cm / 300 mA 40-60 min	Phosphate buffer saline	SYBR Gold	[71]
	Between lysis and electrophoresis, samples were treated with three enzymes ((i) DNA glycosy-lase (FPG); (ii) endonuclease III (endoIII); (iii) T4 endonuclease V (T4endoV))					
Tear duct	Cells, suspended in 0.5% low melting point agarose, were transferred to a slide precoated with normal melting point agarose	pH = 10 2.5 M NaCl 10 mM Tris 100 mM Na_2_EDTA 1% Triton X-100 10% DMSO	pH = 13 300 mM NaOH 1 mM EDTA 20 V/300 mA 20 min	pH = 7.5 0.4 M Tris	Ethidium bromide	[72]

For sample preparation, the cells existing in the tear film that serves as a physiological solution do not need special preparation, while the samples that are contained in the capillary tubes need to be mixed with LMPA (0.5%). To perform the comet assay, the cell mixtures (tears and LMPA) are sampled onto a normal precoated agarose slide and immediately covered with a cover-glass to form a microgel to allow the agarose to jellify. A third LMP (0.5%) agarose layer is added, followed by the immersion of the slides in a lysis solution (pH 10). It is still required to let DNA unwind in electrophoresis buffer and to carry out electrophoresis at 0.8 V/cm [36,72].

**Table 3:** Some methods for isolation and separation of cells from different ocular tissues used for further comet assay studies.

Cell Type	Procedure	Ref.
Corneal Epithelium (porcine)	Trypsin treatment (0.25% trypsin/EDTA): dissected cornea was immersed in trypsin/EDTA, incubated at 37 °C (5% CO2 atmosphere) and released cells from corneal epithelium were collected after 30, 60 or 90 min, depending on the layer needed for each assay. Cell membrane integrity was assessed with the trypan blue exclusion test.	[66]
Corneal(rabbit)	The eyes were enucleated and immersed in PBS. Cells were obtained after enzymatic treatment with collagenase. The cornea was dissected in half, and the anterior section was treated with collagenase to separate the stroma from epithelial cells. With this process, the intact epithelium can be peeled off from the stroma. The epithelial piece was washed with serum-free culture media, and after mincing, was treated with 0.25% trypsin in calcium-free buffer solution.	[73]
Cornea and Retina (Wistar rats)	Dissected tissues were immersed in HBSS buffer (4 °C). A cell suspension was obtained by mincing cornea and retina tissues, in the same buffer, with a tweezer.	[68]
Lens (human)	Cell suspension of single cells was obtained by mechanical disruption. Lenses were removed by an experienced surgeon after corneal incision and were kept in DMEM⁄F12 with 15% FBS and antibiotics at 4 °C. The lenses were then subjected to several rounds of pipetting and the cells were easily obtained.	[71]
Tear Duct	Cells were obtained from tears via collection with a capillary, which contains the exfoliated epithelial cells.	[72]

Abbreviations: EDTA—ethylenediaminetetraacetic acid; PBS—phosphate buffer saline; HBSS—Hank’s balanced salt solution; DMEM—Dulbecco’s modified eagle medium; FBS—fetal bovine serum.

Cerium oxide nanoparticles, known for their antioxidant properties, have been proposed for the treatment of cataracts [70],—an eye condition associated with changes in the structural proteins of the lens and intimately related to other age-related neurodegenerative diseases such as Alzheimer’s [74]. The risk of DNA damage has been evaluated on cultured human lens epithelial cells using the alkaline comet assay. Cells were exposed to 5 and 10 µg/mL of nanoparticles, which did not show any DNA damage or significant increases in the number of sister chromatid exchanges. The reported results confirmed that cerium oxide nanoparticles do not compromise the eye lens and can be exploited in non-surgical cataract treatments.

Guo et al. evaluated the risk of genotoxicity of zinc oxide nanoparticles in rat retinal ganglion cell (RGC-5) lines [75]. The comet assay provided evidence that such nanoparticles are cytotoxic to RGC-5 cells. The integrity of the nuclei was compromised upon cell treatment with zinc oxide nanoparticles, while untreated cells kept intact their nuclei. The extent of DNA damage was shown to be irreversible and concentration-dependent (2.5, 5 and 10 µg/mL). 

Coelho et at. developed therapeutic contact lenses from bacterial cellulose, coated with an organic-inorganic hybrid compound containing aluminum alkoxide and glycidoxypropyl trimethoxysilane, or chitosan nanoparticles to achieve full transparency of the material, which were tested for their risk of genotoxicity using the comet assay in a Chinese hamster ovary (CHO-K1) epithelial cell line [76]. The coating of lenses with organic–inorganic hybrid complex and chitosan increased lens transparency and did not cause any genotoxicity effect; the genotoxicity reported in their studies was attributed to the presence of sodium diclofenac—requiring further confirmation studies.

## 4. Conclusions

Single-cell electrophoresis (“comet” assay) is currently being used as a standard approach for the detection of DNA damage. In this microgel electrophoresis technique, cells are suspended in a thin agarose gel on a microscope slide, are lysed, then electrophoresed and stained with a fluorescent dye that binds to DNA. The cells that have been damaged in their DNA show an increased migration of chromosomal DNA from the nucleus to the anode, resembling a comet. In the alkaline version (the most used), DNA strands break and alkali-labile sites become visible, hence, the amount of migrated DNA quantified is the amount of damaged DNA. Although not routinely used, the comet assay in epithelial cells can be an important tool for risk assessment and also for the diagnosis and prognosis of diseases. Due to the environmental exposure of the ocular mucosa, the use of its epithelial cells may be a straightforward approach in the risk assessment of environmental and occupational exposure besides therapeutic interventions, stimulating the use of the comet assay as a suitable human biomarker. This comet assay has been employed in molecular epidemiology, mainly as a robust biomarker of the early effects of diseases on human populations. The most noticeable advantage is its applicability in almost any cell type, allowing exploration for the use of other biomatrices, including epithelial cells. Epithelia are layers of cells that either line the walls of cavities or channels. Histological analyses have shown that normal tissues containing epithelia are structurally similar. The fact that it is possible to obtain epithelial cells by biopsies or less invasive procedures is an added-value in using such types of cells to evaluate DNA damage safely and economically. As epithelia are in contact with exogenous and endogenous damage sources, this makes them an interesting biomatrix for the individual genotoxicity assessment of many compounds. Besides, the methodology can be used for diagnostic purposes in clinical settings, offering a potential use in patients over time. The studies of lens and corneal epithelial cells present clinical applications, mainly as biomarkers for genotoxicity assessments in human monitoring. Some studies have shown that the lens cells are instrumental for DNA damage detection in individuals with cataracts. This pathology is due to the oxidative stress and UV radiation exposure. The comet assay in ocular cell lines can be used to understand eye pathologies (e.g., macular degeneration). Corneal cells have been used to evaluate DNA damage in cells with the potential to be transplanted, but it is necessary to take into account that additional damage can be induced by the manipulation. Although not commonly used for genotoxicity evaluation, epithelial cells may be an important matrix as the majority of human tumors come from epithelial tissues and the detection of DNA damage can be performed simply by the use of the comet assay.

## Figures and Tables

**Table 1:** Ocular cell lines.

Ocular Cell Line	Characteristics	Refs
RPE-J	RPE-J is a retinal pigment epithelial (RPE) cell line, obtained from the primary cultures of RPE cells obtained on 7-day-old Long–Evans rats.	[37]
RF/6A	RF/6A—monkey endothelial cell line—is spontaneously transformed at an early age and had been passaged over 540 times.	[38]
BCE C/D 1-b	BCE C/D-1b—cow endothelial cell line—is established from explants of normal adult bovine corneas.	[39,40]
WERI-Rb-1	WERI-Rb-1 is a human retinoblastoma tumorigenic cell line used in cell differentiation and biomedical studies, and in animal models of tumor therapy.	[41,42,43]
B-3	B-3 cell line was removed from a human lens obtained from a 5–12-month-old patient who was treated for premature retinopathy. This cell line was infected with Ad12-SV40 at 60% confluence and passage 3.	[44,45]
MP65	MP65 cell line-primary tumor of uveal melanoma of the eye in adults—is part of a panel that imitates the genetic alterations and mutations of this disease.	[46]
2.040 pRSV-T	2.040 pRSV-T cell line is a primary culture of normal corneal epithelium, it is immortalized by transfection with the plasmid pRSV-T using lipofectamine. pRSV-T incorporates the SV40 early region genes and the Rous Sarcoma virus long terminal repeat.	[47]
HCE-2 (50.B1)	HCE-2 cell line is a primary culture of normal corneal epithelium, which by incubation was immortalized with the Ad12-SV40 hybrid virus.	[47]
MP46	MP46 is a cell line from the primary tumor of uveal melanoma of the eye in adults with the same characteristics of the MP65 cell line.	[46]
SIRC	Statens Seruminstitut Rabbit Cornea is a cell line from the cornea of rabbits. The early appearance of distinct cytopathic changes makes it suitable for both the propagation and quantification of the rubella virus, making this cell line appropriate for primary isolation of the rubella virus.	[48]
Fugu Eye	Fugu eye cell line was created from normal eye tissue. These cells maintain a compact genome size with small introns, that are telomerase positive. The cell line can be used for in vitro vertebrate genome research and for marine fish or aquaculture studies.	[49]
ARPE-19 HPV-16	ARPE/HPV-16 transformed cell line was derived from the ARPE-19 cell line by transfection with DH5-HPV-16. ARPE-19 is a spontaneously arising retinal pigment epithelium (RPE) cell line that derived from the normal eyes of a 19-year-old male who died from head trauma in a motor vehicle accident. The cells express the RPE-specific markers CRALBP and RPE-65.	[50]
MP38	MP38 is a cell line from the primary tumor of uveal melanoma of the eye in adults. It belongs to a unique and first panel of 6 uveal melanoma cell lines from either patient tumors or patient-derived tumor xenografts (PDXs). All these cell lines display GNAQ or GNA11 activating mutations. Four of them present BAP1 (BRCA1 associated protein-1) deficiency, which is known to be a characteristic of aggressive disease.	[46]
10.014 pRSV-T	10.014 pRSV-T cell line—an epithelial cell—was transfected with a plasmid that has the SV40 early region primary culture of normal corneal epithelium, using lipofectamine to immortalize the transfection with plasmid pRSV-T. This plasmid has the SV40 early region genes as well as the Rous Sarcoma virus long terminal repeat.	[51]
ARPE-19	ARPE-19 cell line—a transfection host—is a spontaneously arising retinal pigment epithelium (RPE). These cells form stable monolayers, exhibiting morphological and functional polarity. ARPE-19 expresses the RPE-specific markers CRALBP and RPE-65. These cells are diploid and can be carried for over 30 passages.	[50,52]
MP41	MP41 is also a cell line from the primary tumor of uveal melanoma of the eye in adults and has the same characteristics as MP65, MP46 and MP38 cells lines.	[46]
Y79	Y79 cell line was isolated after the enucleation of a primary tumor from the right eye, creating a culture. It is known that the donor had a family history of retinoblastoma. After the development of this culture, it was possible to observe analogous ultrastructural characteristics as the original tumor (mainly nuclear membrane infoldings, triple membrane structures, microtubules, large coated vesicles, basal bodies and centrioles, and annulate lamellae).	[43,53,54,55,56]
MM28	MM28 is also part of a panel of cell lines from the primary tumor of uveal melanoma from the adult eye, from either patient tumors or patient-derived tumor xenografts, also showing GNAQ or GNA11 activating mutations and BAP1 deficiency.	[46]
